# Harmonising ctDNA Measurement in Haematological Malignancies: Traceability, Commutability and Reporting

**DOI:** 10.3390/diagnostics16071056

**Published:** 2026-04-01

**Authors:** Sapha Shibeeb

**Affiliations:** School of Health and Biomedical Sciences, RMIT University, P.O. Box 71, Bundoora, Melbourne, VIC 3083, Australia; sapha.shibeeb@rmit.edu.au

**Keywords:** circulating tumour DNA, haematological malignancies, metrological traceability, commutability, reference materials

## Abstract

Circulating tumour DNA (ctDNA) assays are increasingly applied in haematological malignancies for non-invasive genotyping, quantitative response assessment, measurable residual disease (MRD) detection, and relapse surveillance, often complementing bone marrow-based testing and, in selected scenarios, potentially reducing its frequency. Yet, translating ctDNA results into comparable clinical decisions across laboratories, platforms, and time remains challenging because ctDNA measurements are influenced by the definition of the measurand (for example, variant allele fraction versus mutant molecules per mL), pre-analytical variables, end-to-end workflow losses, and lineage-specific confounders such as clonal haematopoiesis of indeterminate potential (CHIP), therapy-related clonal haematopoiesis, and compartmental disease (marrow, plasma, cerebrospinal fluid, extramedullary sites). This review proposes a harmonisation framework for haematological ctDNA based on three linked concepts—metrological traceability, which connects reported values to reference systems with stated uncertainty, commutability, which ensures that reference materials behave like patient specimens across diverse workflows and fit-for-purpose reference materials that support calibration, and quality control, external quality assessment, and cut-off setting for intended uses such as early molecular response in large B-cell lymphoma, molecular MRD in acute myeloid leukaemia, and deep response monitoring in multiple myeloma. This framework is accompanied by harmonised CHIP-aware reporting rules for settings without matched cellular DNA and practical change-control/bridging strategies to preserve clinical decision thresholds when platforms or bioinformatic pipelines evolve.

## 1. Introduction

Circulating tumour DNA is the tumour-derived fraction of plasma cell-free DNA (cfDNA), inferred through somatic variants, clonotypic rearrangements, copy-number alterations, and/or fragmentation patterns, and is transforming the management of haematological malignancies by providing a non-invasive window into the molecular landscape of disease [1]. In clinical practice, these assays are increasingly utilised for real-time genotyping, quantitative response assessment, and MRD detection, though reported values reflect both tumour biology (shedding, burden, response) and measurement processes (pre-analytics, extraction efficiency, sequencing, bioinformatics) [2]. These applications are particularly useful in the diagnosis and monitoring of haematological malignancies, for example, in leukaemias, where MRD informs risk-adapted therapy; in lymphomas, where ctDNA captures total body tumour burden; and in multiple myeloma, where it offers a blood-based alternative to frequent bone marrow biopsies [3,4,5].

Despite these opportunities, the transition of ctDNA from a research tool to a standardised clinical diagnostic approach is constrained by limited inter-assay and inter-laboratory comparability [6]. Currently, a positive MRD result or a specific variant allele fraction (VAF) in one laboratory may not reflect the same clinical reality in another. This portability gap is driven by the intrinsically multi-step nature of ctDNA testing, in which the reported value reflects not only tumour biology but also pre-analytical handling (tube type, processing delays, plasma volume), extraction and library conversion efficiencies, sequencing depth and error suppression strategies, and bioinformatic evidence rules for calling low-level variants [7]. In haematological malignancies, additional biological confounding further challenges portability, particularly clonal haematopoiesis of indeterminate potential (CHIP) and related age-associated clonal processes that can generate plasma variants unrelated to tumour burden, yet indistinguishable without matched cellular DNA or robust filtering strategies [8].

To achieve the level of harmonisation required for safe clinical adoption, ctDNA testing needs a shared measurement language so that results mean the same thing across platforms. Comparability does not require methodological uniformity. Instead, it depends on different technologies being aligned through the principles of metrology; by defining the measurand and characterising analytical uncertainty, ctDNA outputs can be interpreted consistently across laboratories and clinical settings. This review presents a metrological approach to bridge the portability gap and support standardised ctDNA quantitation in haematological malignancies. Specifically, this review will (i) classify ctDNA measurands and their unit-dependent uncertainty structures; (ii) propose three traceability models (molecule counting, benchmarking truth sets, and clinical decision traceability) tailored to intended use; and (iii) translate these concepts into practical guidance on commutability, reference materials, and a minimum reporting dataset for haematology-focused ctDNA.

## 2. Methods

This narrative review synthesises factors that limit inter-laboratory comparability (“portability”) of ctDNA testing in haematological malignancies, with emphasis on metrological traceability, commutability, reference materials, and clinically portable reporting. Literature was identified through structured searches of PubMed/MEDLINE, Scopus, and Web of Science from January 2010 to March 2026, supplemented by targeted citation chasing of key consensus papers and consortium outputs. Search terms combined ctDNA and haematological malignancy with measurement science and harmonisation terms, including: ctDNA OR cfDNA OR liquid biopsy AND haematology OR leukaemia OR lymphoma OR myeloma AND MRD OR molecular residual disease AND traceability OR metrology OR ISO 17511 OR commutability OR EP14 OR reference material OR calibrator OR external quality assessment OR EQA OR standardisation OR harmonisation OR reporting OR uncertainty.

Evidence was prioritised from (i) expert consensus recommendations and professional guidance documents (e.g., ELBS workshop outputs), (ii) multi-laboratory validation studies and community benchmarking efforts (e.g., BLOODPAC), (iii) analytical validation and quality control frameworks relevant to low-burden detection (including CLSI concepts where applicable), and (iv) studies evaluating pre-analytical variables, assay calibration/quantification units, and bioinformatic pipeline effects on detection capability near decision thresholds. Inclusion focused on English-language publications addressing ctDNA analytical performance, reporting, commutability, traceability models, reference resources, or inter-laboratory comparability; conference abstracts without full methods, single-case reports, and studies lacking sufficient assay design/performance context were not used as primary evidence for harmonisation recommendations. Evidence was synthesised qualitatively and mapped into (a) ctDNA measurands and units, (b) three practical traceability models (Models A–C), (c) commutability considerations for ultra-low burden settings, and (d) a proposed minimum reporting dataset to support clinically portable interpretation across platforms and laboratories.

## 3. Technical Challenges in ctDNA Harmonisation

### 3.1. Standardising the Measurand for Clinical Comparability

A prerequisite for harmonisation is a clear definition of the measurand. In ctDNA testing, multiple measurands are often used interchangeably (Table 1), including, VAF for a specific variant, absolute concentration measures, such as mutant molecules per millilitre of plasma (molecules/mL) or genome equivalents per millilitre (GE/mL) [9,10], global tumour signal metrics, including tumour fraction or tumour-in-normal estimates across a panel, and composite outputs, such as an MRD score or a binary detected/not detected call [11]. Each measurand implies different calibration requirements and uncertainty structures, and therefore different approaches to establishing traceability. For example, VAF is dimensionless and locus-specific, whereas molecules/mL depends on processed plasma volume and end-to-end recovery (extraction and library conversion) [12]. Composite scores (e.g., tumour-informed MRD) can be anchored to reference datasets or decision frameworks but may not map cleanly to SI units [9].

Despite rapid innovation, ctDNA quantitation remains difficult to harmonise because reported values reflect a multi-step workflow (pre-analytical, extraction, library preparation, sequencing, and bioinformatics) in which small differences can yield divergent results near the limits of detection [13]. In haematological malignancies, additional factors further challenge comparability, such as high and variable background leukocyte-derived DNA (particularly with processing delays or leukocytosis), lineage-specific clonal haematopoiesis and therapy-related clonal expansions that can mimic residual disease, disease compartmentalisation (marrow-dominant disease, circulating blasts, nodal/extranodal masses), and treatment effects (e.g., tumour lysis, cytopenias) that alter cfDNA kinetics and the effective denominator for fraction-based measures [14,15]. These features increase the risk that the same numeric result or MRD classification carries different meaning across laboratories, unless the measurand, evidence rules, and performance context are explicitly defined.

In acute leukaemias, MRD informs risk-adapted therapy, transplantation strategies, and post-treatment surveillance [16]. In lymphomas, plasma ctDNA can reflect total-body tumour burden and early molecular response, complementing imaging [17]. In multiple myeloma, deep response assessment increasingly relies on high-sensitivity assays, motivating blood-based approaches to complement bone marrow evaluation [18]. Across these settings, clinical decisions may depend on thresholds close to analytical limits, making transparent performance claims and uncertainty reporting essential for safe interpretation.

Clinical decision thresholds add a further layer of complexity. In baseline genotyping, the primary decision may be whether a variant is present above a calling threshold [19], whereas in MRD assessment the decision rule may integrate multiple variants, longitudinal kinetics, and tumour-informed priors [20]. Accordingly, harmonisation should not force all assays into a single output format; rather, it should ensure that each reported output is well-defined, accompanied by fit-for-purpose performance characteristics and uncertainty, and comparable within its intended clinical use.

### 3.2. Impact of Biology and Pre-Analytics on Low-Burden Detection

**Figure 1:**
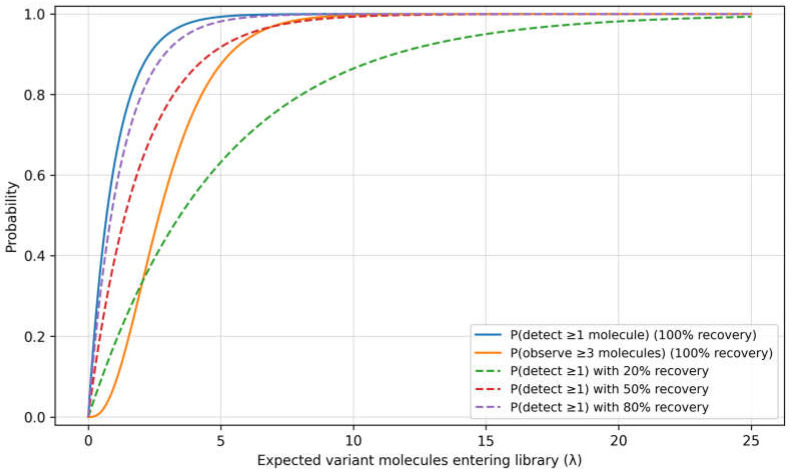
Poisson sampling and detection probability at ultra-low burden. Probability of detecting variant molecules as a function of the expected number entering the sequencing library (λ). Curves show detection of ≥1 molecule at varying recovery efficiencies (100%, 80%, 50%, 20%) and detection of ≥3 molecules at 100% recovery. In the low-burden regime (λ < 3), detection is governed by Poisson sampling, such that small changes in plasma input or recovery can shift results across the limit of detection.

Unlike many serum analytes, ctDNA originates from heterogeneous biological processes that vary with tumour type, burden, vascularity, cell death mechanisms and treatment [21]. Plasma cfDNA also contains a large background from haematopoietic sources, and this background can change with infection, inflammation, trauma, exercise, and pregnancy [22]. At low tumour fractions, biological stochasticity is amplified by pre-analytical effects, such as tube type and stabilisers, time to plasma separation, shipping conditions, and extraction method, which can shift yield and fragment-size distributions [23]. In this low-burden regime, modest changes in background cfDNA or recovery can shift results across the limit of detection, altering both quantitative values and binary MRD calls. This sampling limitation is illustrated in Figure 1.

Pre-analytical guidance developed for specific clinical applications (e.g., plasma-based EGFR testing) underscores that reliable ctDNA testing requires control of the full end-to-end workflow, not only the sequencing or PCR step [21]. Broader cfDNA consensus recommendations similarly emphasise that upstream handling can be a dominant source of variability, particularly in low-burden settings where small shifts in background cfDNA or recovery translate into large differences in apparent ctDNA signal [24]. This has direct implications for harmonisation; fraction-based measurands such as VAF are especially sensitive to denominator fluctuations, whereas volume-normalised reporting such as molecules/mL can be more stable when paired with explicit documentation of processed plasma volume and recovery assumptions. At a minimum, cross-laboratory comparability requires consistent reporting of tube type, centrifugation time and conditions, processed plasma volume, storage/freezing requirements, and extraction method.

### 3.3. Cumulative Error Across the Multi-Stage Analytical Workflow

ctDNA assays operate as composite measurement systems in which losses and biases accumulate across the testing process, including plasma preparation, extraction and quantification, library construction and target enrichment, sequencing and interpretation and reporting [25]. At each step, systematic biases (e.g., GC/sequence-context effects and enrichment-related heterogeneity), random errors (molecular sampling and finite read depth), and classification uncertainty (variant calling, filtering, interpreting and reporting conventions) are introduced [26]. Cross-platform studies indicate that assay sensitivity and reproducibility can diverge at sub-percent VAF levels, depending on input DNA quantity and the chosen error-suppression strategy [26,27]. Harmonisation efforts therefore need to delineate the contribution of each stage to overall uncertainty and make this uncertainty explicit in how ctDNA results are reported.

### 3.4. Intended Use and Performance Requirements

A practical way to operationalise harmonisation is to define performance targets by intended use, recognising that different clinical scenarios demand different balances of sensitivity, specificity, quantitation, and reporting conventions. For example:

- Baseline genotyping for haematological malignancies typically prioritises high specificity for clinically actionable variants (e.g., FLT3, IDH1/2, TP53), clear reporting of genomic regions interrogated, and transparent limits of detection and coverage gaps [19,28].
- MRD surveillance (AML/ALL) or post-therapy lymphoma monitoring demands ultra-low detection capability, robust safeguards against false positives (including explicit management of CHIP/therapy-related clones), and reporting that conveys residual uncertainty near decision thresholds [29].
- Early molecular response (large B-cell lymphoma) benefits from quantitative trend tracking and strong analytical consistency across serial draws; multi-feature approaches (e.g., mutation signal plus fragmentation or panel-level signals) can stabilise measurement at very low tumour fractions [30,31].
- Deep response monitoring (multiple myeloma) requires clear alignment with marrow-based MRD methods, explicit recognition of compartmental disease, and careful interpretation of what a negative blood result can and cannot exclude [32].

Across these clinical scenarios, the decision context determines which error sources matter most and what level of uncertainty is acceptable; therefore, harmonisation should target the decision-critical components of the workflow (pre-analytics, calibration/controls, and evidence rules) rather than forcing all assays into a single reporting format.

## 4. Metrological Traceability for ctDNA: Principles and Practical Models

### 4.1. Applying ISO 17511 Traceability to ctDNA Measurands

International Organisation for Standardisation (ISO) standard 17511 defines requirements for establishing metrological traceability for values assigned to calibrators, trueness controls and human samples measured by in vitro diagnostic medical devices [33,34]. Traceability is achieved through an unbroken calibration hierarchy, with each step contributing to measurement uncertainty, extending to the highest available reference system component (ideally a higher-order reference measurement procedure (RMP) and certified reference material (CRM)) [34]. In ctDNA, this ideal is challenging because many measurands (e.g., variant-specific VAF in fragmented cfDNA in plasma) lack universally accepted RMPs and CRMs. However, traceability can still be strengthened by anchoring discrete components (e.g., copy number concentration) to SI-traceable enumeration methods and documenting the calibration hierarchy and uncertainty contributions end-to-end. Pragmatically, digital PCR (including ddPCR), when partition volume and counting statistics are well characterised, can serve as a candidate reference procedure for assigning mutant and wild-type molecule counts for well-defined variants providing a transparent quantitative anchor when coupled with commutable materials and uncertainty-aware reporting [35,36].

### 4.2. Traceability Models for ctDNA Assays

Multiple calibration hierarchies or traceability models have been described for different measurands, and ISO 17511 explicitly allows traceability to extend to the highest available reference system component when higher-order RMPs/CRMs are not available [33,37].

#### 4.2.1. Model A: Variant-Specific Molecule Counting

A processed reference material (ideally matrix-matched/commutable) is value-assigned for mutant and wild-type molecule concentrations using a digital counting method (e.g., ddPCR), with an accompanying uncertainty statement. Routine assays then calibrate, verify bias, or monitor drift against this anchor—most naturally for targeted assays (ddPCR or small panels) and for anchor variants embedded within broader NGS panels. This model is strongest when partition volume, Poisson/counting assumptions, and reporting essentials are controlled and transparent [36,38].

#### 4.2.2. Model B: Panel-Level Truth Sets and Characterised Datasets (Harmonisation by Benchmarking)

When a single SI-traceable value is not feasible for a composite workflow, the reference can be a characterised sample set and/or dataset spanning variant types (SNVs/indels/fusions/CNV surrogates where applicable) and a defined VAF spectrum. Assays are compared using concordance and error-profiling metrics (sensitivity/precision/false positives by context), as in multi-site cross-platform evaluations and consortium benchmarking efforts. This approach supports cross-platform comparability through harmonised performance claims and shared evidence rules, even when outputs cannot be reduced to a single calibrator value [39,40].

#### 4.2.3. Model C: Clinical Decision Traceability (Traceability to a Decision Framework)

For composite outputs (for example, tumour-informed MRD detected/not detected or an MRD score), the most meaningful traceability target may be the decision framework: the reproducible relationship between assay output, controls, and clinically validated decision thresholds established in trials. Harmonisation then focuses on reproducible score calculation, robust controls and defined acceptance criteria, and explicit transfer/bridging requirements when platforms, panels, or pipelines change [11]. Practically, threshold transfer should be treated as a formal change-control exercise: laboratories should pre-specify the invariant elements (intended use, measurand/unit, positivity rule), run parallel testing on a representative bridging set enriched around the clinical decision boundary (including borderline/near-LoD samples), quantify systematic bias and dispersion between the legacy and new method, and then either (i) retain the existing threshold (if equivalence criteria are met), (ii) recalibrate the threshold, or (iii) apply a documented conversion/bridging rule. Pipeline and panel versions should be locked and disclosed, with post-change QC/EQA monitoring to detect drift over time.

### 4.3. Measurement Uncertainty and Decision Thresholds

In ctDNA, uncertainty accumulates from stochastic sampling when only a small number of mutant molecules are present in the effective input, extraction and library recovery losses, finite sequencing depth and the performance of error-suppression approaches, and bioinformatic classification and filtering rules [26,41,42]. When the expected number of mutant molecules entering library preparation is small, the probability of sampling zero molecules and therefore returning a false-negative result can be substantial even when downstream analytical steps are otherwise optimal [43,44].

From a clinical perspective, uncertainty matters because decision thresholds can lie close to an assay’s detection capability, where small analytical fluctuations can change interpretation [45]. Best practice therefore includes defining and verifying the detection capability of the limit of blank (LoB, highest expected blank), limit of detection (LoD, lowest level detected with stated probability), and limit of quantitation (LoQ, lowest level quantified with acceptable precision) using recognised guidance and adapting analyses to the discrete, low-copy-count nature of ctDNA measurements [36], reporting confidence intervals or uncertainty bounds for quantitative outputs (particularly for enumeration-based measures), rather than single-point estimates alone and specifying how borderline results are managed [46]. For MRD, where a single positive result may trigger major treatment decisions laboratories should clearly distinguish analytical positivity from clinical recurrence risk, and where feasible, incorporate longitudinal confirmation/trend-based decision rules aligned to intended use [45].

## 5. Commutability of ctDNA Reference Materials

### 5.1. Defining Commutability for ctDNA Controls

Commutability describes whether a processed material behaves like authentic patient samples across a defined set of measurement procedures; that is, whether it preserves the same between-method relationships observed for patient samples [47]. When a material is non-commutable, it can yield misleading estimates of bias, distort determination of decision cut-offs (including LoB/LoD), and create over-optimistic performance claims [48]. ctDNA is unusually high-risk for non-commutability because many controls differ from native cfDNA in fragmentation and nucleosomal patterning, matrix composition and inhibitors, and biochemical features that can influence extraction and library conversion [49,50]. For example, mechanically sheared genomic DNA or plasmid spike-ins may perform adequately in downstream analytical steps yet fail to represent end-to-end recovery and fragment loss during extraction and library preparation [51].

Empirical comparisons of commercial cfDNA reference materials against patient plasma demonstrate substantial differences in fragment profiles, background noise, and apparent analytical performance; critically, no single material matches native cfDNA across all evaluated metrics, and inappropriate material choice can shift estimated LoB/LoD and reduce effective clinical sensitivity [49,52].

### 5.2. Designing Commutability Assessments for ctDNA Controls

The Clinical and Laboratory Standards Institute’s (CLSI) evaluation protocol for assessing commutability (EP14) provides a structured protocol for evaluating the commutability of processed samples (e.g., QC or modified human samples) by determining whether they introduce matrix-related biases relative to native patient samples when two quantitative measurement procedures are compared [53]. In the simplest EP14-style design, a panel of individual patient samples is measured by both procedures to establish the patient–sample relationship, and candidate materials are then assessed using regression-based approaches (commonly Deming regression) and prediction intervals or predefined criteria to determine whether the processed material behaves like patient samples [54].

For ctDNA, EP14 designs can be adapted by explicitly defining the measurand (e.g., VAF or molecules/mL), ensuring patient samples span the clinically relevant interval, including near decision cut-offs, and incorporating replicate testing and/or pooled clinical samples when necessary to manage the large random error expected at a low copy number [55].

## 6. Reference Materials, Calibration, Validation, and Benchmarking

### 6.1. Material Classes and Typical Applications

Reference resources used in ctDNA testing span both physical materials and digital benchmarks and can be grouped into five overlapping classes. Importantly, these classes differ in how well they represent native cfDNA properties, and therefore differ in suitability for end-to-end performance, particularly near cut-offs [49].

- Synthetic constructs (oligos, plasmids or synthetic fragments) with predefined variants and mixing ratios. These are convenient, stable and useful for checking analytic steps and calibration of variant fractions, but they generally do not reproduce native cfDNA fragmentation, matrix effects, or extraction recovery [52,56].
- Cell-line-derived DNA mixtures and contrived fragmentation series, often prepared as defined tumour–normal mixtures to create multi-variant panels across a VAF range. These materials support cross-site benchmarking and method comparison but may still deviate from authentic cfDNA in plasma unless explicitly engineered to mimic cfDNA fragment profiles and matrix characteristics [57].
- Human plasma-based materials, including pooled plasma and patient-derived cfDNA. These can better represent the native matrix and cfDNA properties, but are constrained by availability, stability, biospecimen governance, and limited control over variant diversity and concentration ranges [58].
- QC/EQA-oriented materials and multi-laboratory evaluation panels, developed to support routine monitoring, inter-laboratory comparability, and structured analytical validation. In practice, these are often fit-for-purpose rather than universally commutable and should be interpreted in the context of the specific claim being made (e.g., precision monitoring vs. clinical cut-off transfer) [59].
- Digital reference resources and datasets, including curated truth sets, simulated or in silico spike-ins, and community benchmark datasets/call sets used to evaluate bioinformatics pipelines and harmonise evidence rules. These are especially valuable for isolating algorithmic performance and error modes, but do not substitute for wet-lab commutability when making end-to-end clinical performance claims [57].

It is important to note that no single class is sufficient on its own, whereby robust programmes typically require a portfolio of materials and datasets that collectively interrogate upstream handling and recovery, analytic sensitivity/precision, and bioinformatic classification, especially in low-burden settings where material choice can materially change apparent performance. A practical selection guide is summarised in Table 2.

### 6.2. Assigning Target Values to ctDNA Reference Materials

Value assignment is the formal process of assigning a property value together with its uncertainty to a reference material using a defined measurement procedure and documented traceability and control of production. For ctDNA-focused materials, this is ideally aligned with recognised expectations for reference material production and governance [36,52].

Digital PCR is frequently used for value assignment because it can provide absolute quantification by molecule counting in partitions rather than relying on an external calibration curve, making it well suited for assigning copy number concentration and fractional abundance in nucleic-acid materials [35]. Foundationally, droplet digital PCR implements this by counting target molecules across a large number of volumetrically defined droplets and applying Poisson statistics [60]. However, uncertainty and bias can be introduced by partition volume assumptions and their associated uncertainty, particularly if droplet volume is not independently verified for the system in use [61]. In addition, assay-specific effects can dominate error when cluster separation is imperfect, especially at a low copy number [62].

Accordingly, ctDNA value assignment should be accompanied by clear definition of the variant and sequence context, orthogonal confirmation of assigned values, and an explicit uncertainty budget that includes between-run and between-laboratory components [63].

### 6.3. Homogeneity and Stability at Low Copy Number

Reference materials must be demonstrably homogeneous at the intended unit size and stable under intended storage and transport conditions. ISO guidance on reference material production provides structured approaches to designing and analysing homogeneity and stability studies and to incorporating these components into uncertainty. A key implication for ctDNA materials is that, as the target level approaches the assay’s low-burden regime, apparent inhomogeneity can reflect fundamental sampling variability, unless aliquot size, mixing strategy, and study design are matched to the intended claim [64].

For plasma-like materials, stability assessments should include conditions that realistically occur in laboratory practice and should verify not only concentration but also fragment-size characteristics where they are part of the measurand context. Harmonisation-focused cfDNA workflow syntheses highlight that freeze–thaw limits and upstream handling constraints are commonly recommended because upstream variation can materially affect cfDNA integrity and downstream detectability [65]. Practical cfDNA best-practice resources similarly discuss freeze–thaw effects on cfDNA size distribution and emphasise that the magnitude of the effect can depend on extraction method and handling. More broadly, reviews of cfDNA fragmentomics note that repeated freeze–thaw cycles can reduce cfDNA integrity and alter size profiles, reinforcing the need to test and document these conditions for plasma-based materials intended for low-burden applications [66].

### 6.4. Consortium Evidence and External Quality Assessment Insights

The Sequencing Quality Control Phase II (SEQC2) Oncopanel Sequencing Working Group produced a large multi-site, cross-platform proficiency dataset using tailored reference samples spanning variant allele frequencies, input amounts, and sequencing depths demonstrating how structured reference panels can be used to interrogate multiple stages of the workflow [40,58]. Complementary SEQC2 work has focused on quality-control concepts and measurement performance in ctDNA NGS, supporting the idea that standardised controls and shared frameworks are central to reliable cross-site measurement.

The Blood Profiling Atlas in Cancer (BLOODPAC) has similarly reported multi-site work using contrived ctDNA-like materials and shared datasets to comparatively evaluate liquid biopsy testing procedures and to clarify what contrived materials can (and cannot) support when making analytical claims [56,67]. External quality assessment (EQA) schemes consistently show that routine clinical workflows vary materially across labs, with observed errors and wide dispersion in reported values being more prominent at lower allele frequencies and where pre-analytics and reporting practices differ [68]. The International Quality Network for Pathology (IQN Path) pilot EQA demonstrated high error rates and major reporting shortcomings for low-frequency variants, including frequent failure to document key pre-analytical quantities [69]. More recent national EQA efforts using both high-volume plasma samples and artificial reference plasmas likewise highlight that performance depends on the full workflow, and that mixed material types can expose different failure modes [70,71].

## 7. Harmonised ctDNA Reporting in Haematological Malignancies

A central goal of ctDNA harmonisation is to not only improve analytical comparability, but to also ensure that results are reported in a way that is clinically portable across laboratories and platforms [72]. In haematological malignancies, this requirement is amplified by ultra-low burden measurements where stochastic sampling can dominate, and frequent longitudinal monitoring and disease biology that may decouple plasma ctDNA from marrow. Recent expert consensus reports provide a practical foundation for ctDNA report content and wording, including general recommendations on ctDNA use, with the joint American Society of Clinical Oncology and College of American Pathologists guidance on cfDNA assay validation [73], and European Liquid Biopsy Society (ELBS) [74] expert consensus recommendations focused specifically on the reporting of cell-free DNA results. Emerging tumour-informed MRD validation guidance from the BLOODPAC consortium further reinforces the need to make detection capability, input, and decision rules explicit in the MRD setting [11,75].

To operationalise these recommendations for portability, ctDNA reports should explicitly capture pre-examination variables (collection and handling) consistent with laboratory quality frameworks, because upstream handling can materially influence cfDNA background and recovery at an ultra-low burden [76]. In parallel, reports should standardise interpretive language by separating the analytical call (e.g., detected/not detected/indeterminate; quantifiable vs. <LoQ) from clinical inference, which becomes particularly error-prone near the detection boundary [77]. To operationalise clinically portable ctDNA results, a proposed minimum reporting dataset (Table 3) adapted from ELBS expert consensus recommendations and BLOODPAC consortium guidance on lexicon, analytical validation, and reference materials.

Portability also requires that laboratories communicate sample-specific effective sensitivity, because a negative result depends not only on the assay’s nominal LoD but also on the number of informative molecules interrogated (influenced by processed plasma volume, recovery, and molecular depth) [78]. In addition, reports should also present detection capability using established LoB/LoD/LoQ concepts and clearly state the positivity rule and borderline management approach [79]. Precision expectations for low-level quantitation and longitudinal comparability can be anchored to standard precision evaluation principles.

Final reports should include disease-specific interpretive flags that reduce misclassification risk. In particular, clonal haematopoiesis (including therapy-related clonal expansions) can generate variants that mimic tumour-derived signal, especially in MRD-like settings [80,81]. Furthermore, reports should state the CHIP mitigation strategy used and the residual risk when CHIP-prone genes are implicated [82]. For serial monitoring, reports should include assay identifiers and change-control statements to prevent artificial trends driven by method drift [83]. When matched cellular DNA is not available, reports should apply harmonised evidence rules and standardised interpretive labels to minimise clinical misinterpretation.

### Harmonised Evidence Rules for CHIP-Aware Reporting When Matched Cellular DNA Is Unavailable

In many laboratories, paired leukocyte (matched normal) sequencing is not routinely available. In these settings, harmonisation should prioritise transparent disclosure and a consistent evidence-rule framework for CHIP-prone findings, rather than implying tumour origin beyond the available evidence [29,81,82].

- Matched cellular DNA performed: Yes/No; if No, state the mitigation approach used (e.g., panel-of-normals filtering, CHIP gene-list flagging, recurrent artefact list, orthogonal confirmation).
- Residual CHIP risk statement: required whenever a CHIP-prone gene is reported or when a low-level variant is detected near the detection boundary.
- Reporting intent: explicitly separate the analytical finding (detected/not detected/indeterminate) from clinical inference, particularly in MRD-like settings [74,77].

We recommend standardised interpretive labels that can be applied across platforms:

- Tumour-associated (high confidence): supported by tumour concordance (e.g., prior marrow/tissue genotype) and/or burden-tracking kinetics on serial sampling.
- Indeterminate—CHIP possible: insufficient evidence to assign tumour origin (common when only a single timepoint is available or when findings are in CHIP-prone genes).
- CHIP-likely/clonal haematopoiesis suspected: evidence pattern is more consistent with haematopoietic origin than tumour burden.

Pragmatic evidence rules (to support harmonised reporting without matched normal) include:

- CHIP-likely: variant in a CHIP-prone gene and (i) stable VAF over time without concordant disease dynamics, and/or (ii) present in a validated panel-of-normals/recurrent artefact list, and/or (iii) detected in plasma at low level without corroborating tumour-consistent variants despite adequate input/QC.
- Tumour-associated (high confidence): variant is disease-consistent (hotspot/driver for the diagnosed malignancy) and either concordant with tumour genotyping when available or demonstrates serial change consistent with treatment response/relapse.
- Indeterminate—CHIP possible: apply when gene context suggests CHIP risk, but tumour concordance is unknown; recommend longitudinal confirmation and/or orthogonal verification before triggering irreversible clinical decisions [45,74].

## 8. Conclusions

ctDNA testing in haematological malignancies is moving rapidly from research use toward routine clinical applications such as genotyping, response assessment, and molecular MRD-adjacent monitoring. This review argues that true clinical portability is the ability to interpret results consistently across laboratories and platforms, which will depend on the treating of ctDNA assays as measurement systems. Practical harmonisation therefore requires explicit definition of the measurand and decision rules traceability models that anchor at least some components of the workflow to defensible reference systems with stated uncertainty, commutability evidence showing that calibrators and controls behave like patient plasma across the intended range, and structured reporting that makes detection capability, input limitations, and key haematology-specific confounders. Progress will be accelerated by reference materials and external quality assessment programmes designed for ultra-low burden regimes and by disciplined change-control for longitudinal monitoring. Together, these steps provide a realistic pathway to comparable ctDNA results and safer, more consistent clinical decision-making.

## Figures and Tables

**Table 1:** Common ctDNA measurands, example units, and implications for traceability and reporting.

Measurand	Primary Unit	Typical Use	Key Challenges	Harmonisation Strategies
Variant allele frequency (VAF)	% or fraction	Genotyping; resistance tracking	Variable denominator (background cfDNA); CHIP/germline; calling thresholds	Define measurand and calling rules; explicit CHIP handling; report LoD/LoQ + pipeline version
Absolute variant concentration	Molecules/mL plasma (or copies/mL); GE/mL	Response tracking; MRD trending	Recovery (extraction/library); plasma volume; low-level sampling noise	Track recovery (internal standards); volume-based reporting; commutable end-to-end controls
Tumour fraction/signal score	% or score	Lymphoma burden; relapse prediction	Model dependence; panel bias	Shared reference datasets; cross-site validation; standardised model reporting
MRD status (detected/not detected)	Binary call	Clinical decisions; trial endpoints	Evidence-rule dependence; sampling limits; contamination	Standardise evidence rules; report clinical LoD; EQA near cut-off; interpretive language
Clonotype quantitation (IG/TCR)	Molecules/mL plasma	Lymphoma/ALL MRD	Primer/repertoire bias; SHM; clonotype stability	Harmonise target selection; value-assigned clonotype controls; EQA participation

ALL, acute lymphoblastic leukaemia; cfDNA, cell-free DNA; CHIP, clonal haematopoiesis of indeterminate potential; EQA, external quality assessment; GE/mL, genome equivalents per millilitre; IG/TCR, immunoglobulin/T-cell receptor; LoD/LoQ, limit of detection/limit of quantitation; MRD, measurable residual disease; SHM, somatic hypermutation; VAF, variant allele frequency.

**Table 2:** Reference material selection guide for ctDNA harmonisation tasks.

Material Class	Best Suited For	Strengths	Commutability Risks	Practical Tips
Synthetic fragments/plasmids	Variant calling logic; bioinformatics; wet-lab step checks	Stable; defined variants and ratios	May not mimic extraction, fragmentation or matrix	Use for pipeline regression tests; avoid using alone to claim end-to-end LoD.
Cell-line fragmented DNA in plasma	End-to-end LoD/precision; capture/UMI behaviour	Closer fragment size profile; multi-variant panels	Matrix differs from patient plasma; fragmentation not fully native	Verify fragment distribution; include patient-derived negatives for LoB.
Patient plasma pools (high volume)	Pre-analytics; extraction recovery; matrix effects	Native matrix; realistic inhibitors/background	Limited variant spectrum; ethical/availability constraints	Characterise CHIP background; aliquot to reduce freeze–thaw effects.
Foundation/consortium QCMs	Routine QC; cross-lab comparability; training	Designed for scalability; often well documented	Depends on production method; may not be fully commutable	Use EP14-style commutability checks for the intended measurand.
Digital datasets/truth sets	Bioinformatics benchmarking; software change control	Isolates algorithmic performance; shareable	Does not model wet-lab or pre-analytics	Pair with physical materials; version-lock pipelines; publish parameters.
Clonotype/fusion standards (IG/TR, BCR-ABL1, NPM1) in plasma-like matrix	Haematology MRD-specific calibration and EQA	Directly reflects MRD targets used clinically; supports cross-lab comparability	Amplification bias; fragment size and methylation patterns may differ from patient cfDNA	Use fragmented, plasma-spiked materials and verify commutability across extraction + library methods; include CHIP-like negatives.

**Table 3:** Proposed minimum reporting dataset for harmonised ctDNA reporting (adapted from ELBS and BLOODPAC guidance).

Category	Minimum Element	Rationale
Intended use & assay scope	Intended clinical use (genotyping, MRD, monitoring); targets/regions; variant classes detectable (SNV/indel ± CNV/fusion); reference genome build	Prevents misapplication and supports comparability across assays and laboratories
Specimen & pre-analytics	Specimen type (plasma); tube type; time to processing and centrifugation conditions; key deviations	Pre-analytics affect background and yield; required for reproducibility and cross-site interpretation
Input & quantity basis	Plasma volume processed; cfDNA yield; library input; unit basis (VAF and/or copies/mL)	Supports interpretation of negatives/low-level calls and enables longitudinal comparison
Detection capability & calling rules	LoD/LoQ (or validated detection capability); positivity threshold; handling of borderline results and replicates	Avoids over-interpretation near cut-offs and supports consistent classification between laboratories
Result & uncertainty	Quantitative result (VAF and/or copies/mL) with confidence interval or stated uncertainty; key QC pass/fail indicator	Enables clinically meaningful trending and transparent confidence in the measurement
Confounders & biological limitations (incl. CHIP)	CHIP/therapy-related clone mitigation approach and residual risk; major limitations (e.g., heterogeneity, low shedding)	Reduces false positives/negatives and improves portability of interpretation
Disease compartment context	Relevant concurrent disease context if available (e.g., marrow MRD, imaging response, CNS, or extramedullary involvement)	Interprets discordance when plasma ctDNA under-represents compartmental disease

## Data Availability

No new data were created or analyzed in this study. Data sharing is not applicable to this article.
